# Centering disability in digital health: perspectives and actionable approaches to accessibility

**DOI:** 10.3389/fdgth.2026.1923565

**Published:** 2026-09-02

**Authors:** Hilary Touchett, Kelley Arredondo, Stephanie C. Day, Brenda Salgado, Katherine Bay, Jan Lindsay, Felicia Skelton

**Affiliations:** 1 Center for Innovations in Quality, Effectiveness and Safety (IQuESt), Michael E. DeBakey VA Medical Center, Houston, TX, United States; 2 Department of Medicine, Baylor College of Medicine, Houston, TX, United States; 3 South Central Mental Illness Research, Education, Clinical Center, a Virtual Center, Houston, TX, United States; 4 VHA Office of Rural Health’s Veterans Resource Center, Salt Lake City, UT, United States; 5 VHA Office of Rural Health’s Veterans Resource Center, White River Junction VT, United States; 6 Department of Psychiatry and Behavioral Sciences, Baylor College of Medicine, Houston, TX, United States; 7 Department of Physical Medicine and Rehabilitation, Baylor College of Medicine, Houston, TX, United States

**Keywords:** digital health, disabled persons, health equity, International Classification of Functioning, Disability and Health, universal design

## Abstract

Digital health innovations hold enormous potential to expand access to care. Yet individuals with disabilities often face inequities due to systemic, environmental, and design-related barriers that limit full participation in digital health ecosystems. These challenges often arise from the ways digital tools and systems are designed rather than the impairment itself. This perspective discusses the intersection of disability, digital health, and equitable access through key disability frameworks including the International Classification of Functioning (ICF), the Health Equity Framework for People with Disabilities, and other prominent medical and biopsychosocial models. We assert that equitable digital health for people with disabilities requires a shift toward accessibility-as-standard, grounded in Universal Design, participatory co-design, and intersectionality-informed approaches. We offer applied guidance on integrating Universal Design and Web Content Accessibility Guidelines (WCAG) principles from the earliest design stages; incorporating assistive technology compatibility; developing accessibility informed user profiles; establishing digital literacy and support pathways; adapting telehealth workflows for diverse needs; and building trust through culturally aligned content. We also outline strategies for equitable design such as embedding disability representation in co-design processes and developing measurement plans that capture accessibility, engagement, and long-term outcomes for people with disabilities. Achieving equitable reach and impact in digital health requires centering disability as a core design and implementation domain. By translating conceptual disability models into concrete design practices, and workflow adaptations, clinicians, researchers, and technology developers can create digital health environments that are more usable, inclusive, and sustainable improving access, experience, and health outcomes for disabled individuals across diverse settings.

## 1 Introduction

Digital health tools (DHT) have an increasingly central role in healthcare, serving as a critical mechanism for expanding access and augmenting traditional in-person care (1). DHT encompass a broad range of technologies, including telehealth platforms, patient portals, mobile health (mHealth) applications, remote monitoring services, and asynchronous communication systems. Initially, telehealth was widely promoted as a strategy to increase access to care in remote and underserved communities (2), where geographic distance and provider shortages often limit timely healthcare delivery. Over time, telehealth has demonstrated a range of benefits across diverse populations, including greater scheduling flexibility, convenience, reduced travel burden, lower stress associated with clinic visits, and shorter wait times, while preserving care quality and patient satisfaction.

Despite these benefits, the rapid implementation and integration of DHT raised concerns that digital transformation may inadvertently widen existing health inequities, disproportionately impacting some communities (3). Access to digital health is shaped by internet connectivity and device ownership as well as the accessibility of the technologies themselves and the systems in which they are embedded. For those with intersecting identities with access related barriers (i.e., older adults, individuals with lower incomes, rural residents, individuals with disabilities), digital health may create new forms of exclusion if tools are designed without accessibility in mind (4). The challenge of creating accessible and equitable digital tools and workflows has resulted in delayed access to new health innovations. Individuals who are older, live-in rural areas, and have a disability are less likely to own personal devices and access the internet regularly, creating inequities related to technology and internet access (5, 6).

In the context of disability, unequal use of DHT often results from design, workflow, and system level shortcomings that create barriers to easy and satisfactory use (7). As such, multilevel supports are needed to ensure optimal impact of DHT among those with complex disabilities (e.g., accessible DHT, adaptive supports, and inclusive workflows) (8). In this perspective, we describe the value of a disability inclusive approach in developing and implementing DHT, reviewing key disability frameworks and accessibility principles while providing actionable guidance for designing and incorporating accessible DHT and systems of care.

## 2 Conceptual foundations: disability and digital health

Disability is increasingly understood as a dynamic interaction between health conditions, personal factors, and environmental contexts, not a fixed characteristic of an individual (9). This distinction is especially important for digital health where the design of technologies and care delivery systems can facilitate or restrict participation in care. Although disability is often conflated with physical or mental impairment, theoretical models of function emphasize that disability is the result of the interaction of medical impairment with the personal and environmental characteristics of an individual that create activity limitations that then restrict participation in daily life (10). Therefore, disability is a social construct rather than an absolute; DHT and health systems can either reduce barriers to care or create new sources of exclusion, depending on how they are developed and implemented.

Grounding digital health in disability theory is essential because many barriers encountered in virtual care are not inherent to a person's impairment, instead resulting from inaccessible technologies, workflows, and institutional practices. For example, a patient with low vision may participate fully in telehealth when platforms are compatible with screen readers and accessible navigation tools. The same patient may be effectively excluded when systems rely on unlabeled buttons, inaccessible authentication processes, or visual prompts. This perspective shifts attention away from individual deficits and toward system design as the determinant of equitable participation.

**Figure 1:**
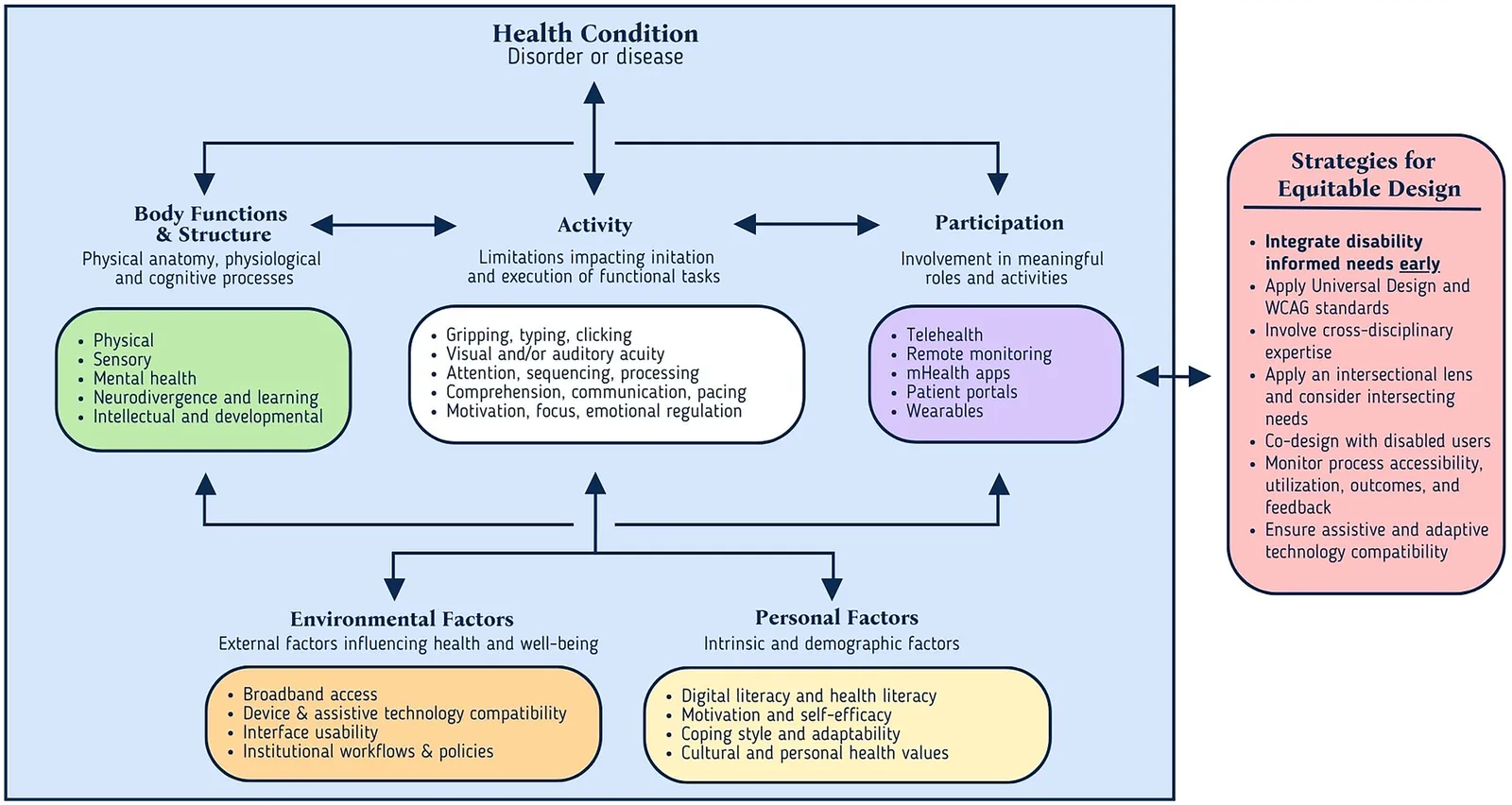
The ICF and digital health. This figure presents a conceptual model based on the International Classification of Functioning, Disability and Health (ICF) framework (10), adapted to illustrate factors related digital health participation and strategies for equitable design.

The World Health Organization's International Classification of Functioning, Disability and Health (ICF) framework provides a useful framework for understanding these interactions. The ICF conceptualizes functioning as the product of relationships among health conditions, body functions and structures, activity, and participation, contextual factors. Activity and participation include two distinct but related components, activity, or the execution of a task or action by the individual, and participation, the involvement an individual has in life situations. Contextual factors include both environmental factors (i.e., physical, social, and attitudinal environments), which can facilitate or hinder engagement in daily life, and personal or identity-related factors (e.g., age, race, socioeconomic status). In digital health, environmental factors can include broadband access, device compatibility, interface usability, and institutional workflows. Applying the ICF to digital care (as seen in Figure 1) highlights how inaccessible digital health technologies can themselves become disabling environments when they restrict an individual's ability to participate in health-related activities.

Furthermore, The Health Equity Framework for People with Disabilities extends this perspective by emphasizing how structural determinants shape health outcomes (11). This framework highlights the role of social policies, institutional practices, and resource allocation in producing inequities. In digital health, these determinants include standards for accessible technologies, reimbursement policies for accessible accommodations, and organizational investment in digital literacy support. Importantly, these are modifiable system-level factors, suggesting that inequities in digital health are not inevitable consequences of disability, but outcomes of design and implementation choices.

Lastly, traditional medical models of disability locate the “problem” within the individual and focus on treatment or remediation of impairment (12). In contrast social models emphasize that external, societal and environmental, barriers create disability by limiting participation. Biopsychosocial models integrate these perspectives, recognizing that health conditions interact with social and environmental contexts to shape functioning. For digital health, this integrated lens is particularly useful, acknowledging individual needs while centering broader systems that enable or constrain access. Taken together, these frameworks underscore a fundamental principle: digital health equity requires shifting responsibility from the individual to the system, ensuring that accessibility is embedded in design, implementation, and care delivery from the onset.

## 3 Accessibility-as-standard: principles and approaches

Achieving equitable digital health requires treating accessibility as a foundational design principle rather than an optional accommodation. Designing for accessibility from the onset can help ensure solutions are effective, complete, and cost-effective while reducing barriers for people with disabilities. Incorporating accessibility considerations early during development improves usability for a broader range of users, including older adults, individuals with limited digital literacy, and those navigating temporary situational constraints.

Universal Design is a central framework for this approach, referring to the creation of products and environments that are usable by the widest possible range of individuals without the need for specialized adaption (13–15). In digital health, Universal Design supports development of technologies that account for diverse physical, sensory, cognitive, and communication needs from the beginning. Examples include patient portals with adjustable text size, telehealth platforms that support captions and multiple communication modalities, and remote monitoring tools that offer flexible methods for data entry. Universal Design shifts accessibility from a niche consideration to a standard expectation, recognizing that inclusive systems benefit all users (16).

Operationalizing Universal Design in digital platforms is often guided by the World Wide Web Consortium Web Content Accessibility Guidelines (WCAG), which provides internationally recognized standards for accessible digital content (17). WCAG is organized around four principles: content should be perceivable, operable, understandable, and robust. Perceivable systems ensure users can access information through multiple sensory channels, such as captions for audio content or alternative text for images. Operable systems allow navigation using a variety of inputs, including keyboards, touchscreens, and adaptive devices. Understandable systems prioritize clear language and predictable interfaces. Robust systems maintain compatibility with current and emerging assistive technologies. Embedding these principles during initial development is substantially more efficient than attempting to retrofit inaccessible platforms after deployment.

Compatibility with assistive technologies is another essential component of accessibility as standard (18). Many individuals rely on aids such as screen readers, refreshable braille displays, eye-tracking systems, or voice recognition software to engage with digital environments. When DHT are designed and tested with these technologies from the outset, users with disabilities are more likely to have consistent and effective engagement in digital healthcare. Routine interoperability testing with commonly used assistive devices should therefore be integrated into design and quality assurance processes.

An accessibility framework must also account for intersectionality. Disability often intersects with age, language, race and ethnicity, socioeconomic status, and geographic location to shape digital access (19, 20). For example, a rural older adult with hearing loss and limited broadband access may face substantially different barriers from those of an urban younger adult with the same impairment. Intersectionality informed accessibility recognizes these overlapping identities and highlights the importance of culturally responsive content, multilingual design, and flexible workflows to address broader structural barriers. Treating accessibility as standard, rather than exceptional, is essential for creating digital health systems that are equitable, inclusive, and sustainable.

## 4 Discussion: actionable strategies for equitable design and implementation

### 4.1 Design principles and practices

Often, digital health design teams address accessibility as a matter of compliance for WCAG at the end of development, rather than centering accessibility from initial conceptualization throughout development (3, 21). WCAG compliance is insufficient for inclusive design because it sets a floor for minimal accessibility rather than the standard. Rather than using standard UX/UI processes to focus on modal users who represent the average customer, centering Universal Design principles ultimately results in more usable systems for everyone. For example:

- High contrast text improves legibility for all users
- Low motor precision requirements improve usability on mobile devices
- Compatibility with screen readers translates to better search engine optimization parsing and comprehension
- Captions and transcripts enhance comprehension in noisy environments for those with hearing loss and second language users
- Voice to text and voice commands enhance handsfree multitasking while working out or walking

In early stages of conceptualization, development of evidence-based, disability informed user profiles for a range of disability domains including: motor impairment (22), visual and auditory impairments (23, 24), mental health contexts (25), neurodivergence (26), and intellectual impairments (27) can aid the team in understanding specific needs. This can be helpful for prototype development before entering the formal co-design workshop, enabling co-designers to have a shared understanding of the goal.

#### 4.1.1 Human-centered, participatory, and inclusive co-design

Human centered, participatory, and co-design approaches ensure people with disabilities can have an active and collaborative role which ultimately translates to higher quality products. Studies show that early inclusion is an effective strategy when developing usable and accessible DHT (4, 28). Representation should leverage community expertise by including individuals with lived experience of the conditions being targeted and the ancillary community (e.g., support people, clinicians, advocacy groups). However, there are many challenges to consider when employing co-design and participatory methods with disability communities. Recruitment can be challenging due to practical barriers such as symptom fluctuations, travel, access, under-representation, or distrust/past negative experience with health and research institutions. Accessibility is often not standard in research processes; online scheduling tools, video conferencing, dense and jargon full consent forms, lengthy workshops may create barriers for participation. Power imbalances can be difficult to manage when co-design groups hold institutionally authoritative roles like researchers, physicians, engineers. Furthermore, implementing insights from co-design can often be challenging, particularly in agile development cycles because complex tasks and long-term projects are often deprioritized in favor of ‘easy wins.’ In fact, many features of a rigorous co-design process (e.g., sustained relationships, accessible processes, intersectional recruitment, meaningful decision-making power) make it time and resource intensive. Additionally, from an equity perspective, disabled communities have historically been over-researched and underserved which underscores the importance of demonstrating and reciprocity to earn trust before asking for participation. Table 1 provides recommended techniques to supporting accessible co-design methods.

**Table 1:** Strategies to support accessible co-design methods.

Approach	Rationale	How to implement
Accessible materials	Different strategies can be used to meet a range of information and communication preferences	Employ audio, visual, and written materials. Ensure consistency across sources. Provide materials in advance
Flexible engagement	Variability in health status may impact participation, flexibility may mitigate drop out and exclusion.	Allow for both synchronous and asynchronous feedback in writing and/or by voice.
Accommodation as default	Offering accommodation as a default reduces the pressure for the individual to articulate their needs	Schedule frequent breaks. Use built-in functions on your platform to provide written transcriptions, audio recordings, and closed captioning.
Compensation for expertise	Inclusion requires support to participate. Free labor may skew towards individuals with the time and means to participant and not represent the end user group accurately.	Providing fair compensation for actual time and effort spent, while employing other strategies (e.g., flexible engagement) to accommodate barriers.
Peer leadership	Centers the project around members of the intended community and promotes true integration of the disability perspective into the process.	Engage active members of the disability community to be a part of the core design leadership team. Provide mentoring/support where needed but also allow room for their knowledge/expertise to shine through.
Psychological safety	Encourages and supports an open dialog across all team members to ensure all points are respected and heard.	Use frequent check ins, explicit rules of engagement and invitations to provide thoughts/opinions even if controversial; use non-involved observers to moderate and ensure all views are heard.
Iterative validation	Holds accountability in progress and alignment when working towards more difficult objectives	Using multiple checkpoints and data streams (e.g., getting feedback from different groups within the community) to ensure the product is widely useful and not overly customized to the exact participants in the co-design group.

### 4.2 Disability centered workflows and implementation considerations

It is important to note that DHT do not function in silos and accessibility needs extend beyond the tool itself. A fully accessible tool implemented within an inaccessible system or workflow still renders the tool ineffective. When developing disability centered workflows, consider key accessibility points such as scheduling, communication modalities, and digital literacy. Many disorders and impairments have unpredictable capacity due to fluctuations in symptoms, fatigue, pain, or care needs. Flexible scheduling and format, maintaining openings for short notice bookings, easy rescheduling, and time-of-day flexibility are essential to facilitate access. Additionally, using multiple communication modalities is crucial to providing access to a range of needs. For example, while video telehealth is often touted as the gold standard for remote care because it provides more clinical information than phone/audio only, using a video telehealth platform without closed captioning can disproportionately impact users who are Deaf or Hard of Hearing, individuals with limited bandwidth, and those who rely on assistive technology incompatible with video telehealth platforms. As a result, patient needs and preferences should be balanced against the need for clinical information when considering modality. Furthermore, clear instructions for preparing for, attending, and following up after appointments are areas of frequent failure. Instructions are often written from the assumption of digital fluency, specific hardware, stable connectivity, and language fluency. Accessibility prioritizes using plain language, using brief, step-by-step and numbered actions, providing written, audio, and video instructions, and explicit information about what to do if something goes wrong.

Moreover, health systems' default assumption is that the end user is independent and self-directed, excluding many who prefer or rely on a support person to manage their health needs. Like applications and web content, processes and workflows should start with an assumption of accommodation as a standard consideration to avoid relying on a patient requesting or knowing how to navigate the system to use accommodations within the system. Frequently system designs center the digital and health literacy objectives around teaching the patient and support system how to navigate their individual processes and systems, but this reflects a system centered schema rather than a disability centered foundation as the ICF and health equity framework would recommend. In short, system design should require minimal prior competence and support, rather than requiring users to learn how to function within the system to optimize accessibility.

However, workflow adaptations and system support are unlikely to be effective without a clearly articulated plan for measurement and accountability to accessibility. Pragmatic implementation and evaluation outcomes should be evaluated and reported with the same rigor as clinical outcomes. Consideration for each level of the program from the accessibility and usability of the DHT, the accessibility of the workflow, engagement and utilization metrics, as well as health outcomes and patient satisfaction over time, are all necessary to evaluate the overall performance. Organizations can support this mission by reporting accessibility metrics along with other pertinent clinical quality indicators and creating feedback mechanisms to iteratively perform quality improvement.

### 4.3 Implications for clinicians, developers, and researchers

Equitable digital health and accessibility are best facilitated when clinicians, developers, researchers, and individuals with disabilities work collaboratively to achieve shared competencies and goals. Awareness of each disciplinary lens (clinicians’ knowledge of patient experiences and care workflows, developers’ knowledge of technical systems and design constraints, and researchers’ expertise in evidence generation, measurement), can aid teams in improved communication, better identification of knowledge gaps, and ultimately more effective collaboration.

Although depth of expertise will vary by role and experience, there are key domains that all team members should have shared foundations in:

- **Knowledge and awareness:** Basic foundations in disability frameworks, Universal Design principles and accessibility standards will help members evaluate design decisions against equity goals. This includes awareness of accessibility features and assistive technologies used in practice, and how intersecting identities shape access and how unconscious bias including within professional and institutional cultures shapes what gets built and for whom.
- **Relational and process accountability:** Involving collaborators with disabilities thoughtfully, compensating their expertise equitably, using a curious and supportive approach, centering accessibility in the co-design process, and fostering long-term mutually beneficial relationships with disability communities are essential team skills.
- **Embedding quality improvement:** Documenting accessibility needs, measuring outcomes rigorously, using findings to iteratively improve each design cycle, advocating within institutional structures for accessibility as standard. Including ensuring this expectation is hard-wired into the process so that it is not left to individual initiative to persist.

## 5 Future directions

Centering disability in digital health requires sustained, systemic changes that permeate beyond individuals, projects, tools, or well-intentioned choices. A major priority should be strengthening the evidence base. Recognizing that the current evidence base is likely built on exclusion or oversimplification of the needs and experiences of individuals with disabilities means that another needed area is finding consensus on preferred outcome measures and validating them for disabled populations. Critically, there is a lack of longitudinal outcome data on disabled patients’ experiences with DHT and the wide variability in this population means more concerted efforts are needed to ensure equitable representation.

At the policy level, institutions and regulatory bodies should commit to reinforcing accessibility as standard in procurement, advocate for reimbursement policy that covers accommodations in telehealth modalities, and expect organizational accountability in tracking accessibility metrics among clinical quality indicators. Cross disciplinary and cross-sector partnerships are needed to ensure ongoing and thoughtful exploration of emerging challenges continues. Disability inclusive standards should continue to be developed. Whereas WCAG and other relevant policies like ADA and 508 compliance set minimum standards, more work is needed to co-design disability inclusive frameworks to guide optimization of accessibility in DHT. Lastly, given the prevalence of both seen and unseen disability in the community, training pipelines like medical schools, health informatics, and UX training should incorporate disability and accessibility curriculum to create lasting systemic change. The overarching message is that these barriers are not the inevitable result of disability; they are the result of design and implementation failures, which means they can be changed.

## 6 Conclusion

Equitable digital health can only be achieved by centering disability throughout the design, testing, and implementation process. This article posits that the barriers individuals with disabilities encounter when using DHT are not inherent facets of disabilities but are produced by poor design across technology, workflows, and institutional practices. By grounding digital health design foundations in disability frameworks, embedding Universal Design and co-design principles throughout the development process, we can create systems that are more usable, equitable, adaptable, and resilient. Building DHT and systems designed to meet the needs of all users ensures that innovative care reaches those who need it most, the ultimate goal in equitable healthcare.

## Data Availability

The original contributions presented in the study are included in the article, further inquiries can be directed to the corresponding author.
